# Complete Genome Sequence of Mycobacterium avium subsp. *hominissuis* Strain JP-H-1, Isolated from an Equine Abortion Case in Japan

**DOI:** 10.1128/MRA.01228-19

**Published:** 2019-11-27

**Authors:** Yuta Kinoshita, Hidekazu Niwa, Eri Uchida-Fujii, Toshio Nukada

**Affiliations:** a Microbiology Division, Equine Research Institute, Japan Racing Association, Shimotsuke, Tochigi, Japan; University of Arizona

## Abstract

Here, we describe the complete genome assembly of Mycobacterium avium subsp. *hominissuis* strain JP-H-1, collected from an equine abortion case in Japan. JP-H-1 has a 5,491,452-bp circular chromosome and 3 plasmids.

## ANNOUNCEMENT

Mycobacterium avium subsp. *hominissuis*, a subspecies of the Mycobacterium avium complex (MAC), is frequently isolated from human and pig mycobacteriosis cases (1, 2). Mycobacterial infections are rarely found in horses, but occasional infections, especially by members of the MAC, have been reported (3, 4). The M. avium subsp. *hominissuis* isolate described here comes from an equine abortion case in Hokkaido, Japan, in 2018.

M. avium subsp. *hominissuis* strain JP-H-1 was isolated from the stomach content of an aborted equine fetus in Japan in 2018. Stomach content (100 μl) was directly inoculated onto Middlebrook 7H11 agar plates (Kyokuto Pharmaceutical Industrial Co., Ltd., Tokyo, Japan) and incubated at 37°C for 2 weeks in a 5% CO_2_ atmosphere. A single colony was picked up and used for the following steps. The isolated strain was incubated in Middlebrook 7H9 broth (Fujifilm Wako Pure Chemical Corporation, Tokyo, Japan) supplemented with Middlebrook ADC enrichment medium. Its genomic DNA was obtained in accordance with a previous method (5), with the addition of a beating step with zirconia beads before the proteinase K process. For Nanopore sequencing, libraries were prepared with a ligation sequencing kit (catalog number SQK-LSK109; Oxford Nanopore Technologies [ONT]), without fragmentation, and sequenced on a MinION sequencer using a FLO-MIN106 R9.4 flow cell (ONT). The same genomic DNA was also sequenced on an Ion Torrent Personal Genome Machine (PGM) instrument (Thermo Fisher Scientific) using the Ion 318 chip v. 2 BC and Ion PGM Hi-Q View Chef reagents. Base calling of raw fast5 data from the MinION was carried out in Guppy v. 3.2.2 software (ONT), specifying the flipflop model. Before assembly, a quality-controlled data set was created in Porechop v. 0.2.4 software (6) with default settings to remove adapters and in NanoFilt v. 2.5.0 software (7) to filter by lengths of ≥1,000 bp and quality scores of ≥8. The genome was assembled from 272,629 high-quality long reads with an average read length of 9,825 bp. *De novo* genome assembly was carried out in Flye v. 2.5 software (8) with its “-plasmids” option, specifying the estimated genome size (-genome-size 5.5 m), number of polishing iterations (-i 3), and Nanopore reads (-nano-raw). Circularity was confirmed by assessing assembly graphs using Bandage v. 0.8.0 software (9). The first polish was run in Racon v. 1.4.3 software (10), using the high-quality long reads three times with the following parameters: score for matching bases (-m), 8; score for mismatching bases, (-x) −6; default gap penalty (-g), −8; and default window (-w), 500. Short reads generated by the Ion PGM were trimmed in Sickle v. 1.33 software (11), using a quality threshold of 25 and a length threshold of 100 bp. The final polish, using the 1,853,619 high-quality short reads whose total read length was 340.8 Mb, was carried out in Pilon v. 1.2.3 software (12) with default parameters.

Our polished assembly consists of a 5,491,452-bp circular chromosome (GC content, 68.9%) and three plasmids ranging in size from 13,441 to 191,151 bp (GC content, 64.4% to 66.3%). The JP-H-1 genome was annotated in DFAST v. 1.2.3 software (13). It contained 5,469 coding sequences (CDSs), 55 tRNAs, and 3 rRNAs.

### Data availability.

These data are deposited in DDBJ/ENA/GenBank under accession numbers AP020326, AP020327, AP020328, and AP020329. Raw reads are available under BioProject accession number PRJDB8716.
